# Comparison of Raw Data-Based and Complex Image-Based Sparse SAR Imaging Methods

**DOI:** 10.3390/s19020320

**Published:** 2019-01-15

**Authors:** Zhilin Xu, Bingchen Zhang, Hui Bi, Chenyang Wu, Zhonghao Wei

**Affiliations:** 1 Key Laboratory of Technology in Geospatial Information Processing and Application System, Institute of Electronics, Chinese Academy of Sciences, Beijing 100190, China; bczhang@mail.ie.ac.cn (B.Z.); cywu1992@163.com (C.W.); weizhh@163.com (Z.W.); 2 Institute of Electronics, Chinese Academy of Sciences, Beijing 100190, China; 3 School of Electronic, Electrical and Communication Engineering, University of Chinese Academy of Sciences, Beijing 101408, China; 4 College of Electronic and Information Engineering, Nanjing University of Aeronautics and Astronautics, Nanjing 211106, China; bihui1991@163.com

**Keywords:** SAR imaging, Lq regularization, azimuth-range decouple, down-sampling, displaced phase center antenna (DPCA), azimuth ambiguity

## Abstract

Sparse signal processing has already been introduced to synthetic aperture radar (SAR), which shows potential in improving imaging performance based on raw data or a complex image. In this paper, the relationship between a raw data-based sparse SAR imaging method (RD-SIM) and a complex image-based sparse SAR imaging method (CI-SIM) is compared and analyzed in detail, which is important to select appropriate algorithms in different cases. It is found that they are equivalent when the raw data is fully sampled. Both of them can effectively suppress noise and sidelobes, and hence improve the image performance compared with a matched filtering (MF) method. In addition, the target-to-background ratio (TBR) or azimuth ambiguity-to-signal ratio (AASR) performance indicators of RD-SIM are superior to those of CI-SIM in down-sampling data-based imaging, nonuniform displace phase center sampling, and sparse SAR imaging model-based azimuth ambiguity suppression.

## 1. Introduction

Synthetic aperture radar (SAR) is a significant remote sensing technology, which has been widely used in various fields, such as marine monitoring, topography mapping and target detection [1,2]. In recent years, sparse signal processing theory has been introduced to SAR imaging, which shows that the sparse observed scene can be reconstructed with less sampled data [3,4,5]. Compared with a matched filtering (MF)-based method, a sparse signal processing-based SAR imaging method can reduce the system complexity and improve the image quality efficiently, addressing noise, sidelobes and azimuth ambiguity suppression [4,6].

Compressive sensing (CS), the recent main achievement of sparse signal processing theory, was firstly introduced to radar imaging by Baraniuk [7]. In 2010, Patel et al. proposed a more general CS-based SAR imaging model to recover observed scenes, and analyzed different azimuth sampling strategies with spotlight SAR data [8]. Cetin et al. explored the principle of autofocusing and moving target imaging based on CS [9]. Zhang et al. achieved resolution enhancement for inversed synthetic aperture radar (ISAR) imaging via CS [10]. Ender analyzed the reconstruction performance of CS-based wavenumber domain imaging algorithms via the ISAR echo data [11]. Chen et al. proposed a parametric sparse representation (PSR) method for motion compensation and refocusing of moving targets [12,13].

Because raw data is coupled in the azimuth and range directions, the sparse reconstruction method based on an observation matrix has huge computational cost, which makes it impossible for large-scale observed scene reconstruction. To solve the above problem, the azimuth-range decouple-based sparse SAR imaging method was proposed in [14]. The method can reduce imaging time efficiently and improve image performance based on the fully sampled or down-sampled echo data for the sparse region [14,15]. The method has been widely applied to ScanSAR [16], TOPSAR [17], Sliding Spotlight SAR [18] and so on. Quan et al. applied sparse signal processing methods to nonuniform displace phase center sampling SAR imaging [19]. The new CS-SAR imaging method using the multiple measurement vectors model was also proposed to reduce the computation cost and enhance the imaging result [20].

Additionally, a sparse signal processing-based imaging method also can obtain feature-enhanced radar images [21]. Samadi et al. developed an image formation technique that simultaneously enhances multiple types of features [22]. In order to further reduce computational complexity, a complex image-based sparse SAR imaging method was proposed [23]. The method uses an MF-constructed complex image as the input, and then obtains a feature-enhanced SAR image by solving an $l_{q}$ regularization problem. It is pointed out that raw data-based and complex image-based sparse SAR imaging methods are equivalent when the data is fully sampled. When the data is down-sampled, they are not equivalent.

Although the complex image-based sparse imaging method (CI-SIM) can reduce computational complexity, the raw data-based sparse imaging method (RD-SIM) can achieve better imaging performance in some cases. It is important to understand the similarities and differences between them for the selection of appropriate algorithms. In this paper, we discuss their relationship and promote the more general conditions of inequality between these two methods. The performance of RD-SIM is superior to CI-SIM when raw data is under-sampled. Under-sampling includes not only down-sampled data-based, imaging which is only mentioned in [23], but also nonuniform displace phase center sampling, sparse SAR imaging model-based azimuth ambiguity suppression and so on. Furthermore, experiments are carried out in full-sampling and three under-sampling cases to verify our conclusion.

The rest of this paper is organized as follows: Section 2 introduces sparse SAR imaging models based on raw data and complex images, respectively. We will analyze their equivalence at full-sampling and the inequality at under-sampling, which provides a theoretical basis for algorithm selection. Sparse SAR imaging with full- and under-sampling is introduced in Section 3. In Section 4, we compare the performance of these two sparse SAR imaging methods under full-sampling and three under-sampling cases. Finally, conclusions are drawn in Section 5.

## 2. Materials and Methods

The baseband signal echo of all targets in the observed scene can be represented as:

(1) $$\begin{array}{l} y\left(t,\tau \right)=\iint_{\left(p,q\right)\in C}x\left(p,q\right)\omega_{a}\left(t-\frac{p}{v}\right)\exp\left\{-j4\pi f_{c}\frac{R\left(p,q,t\right)}{c}\right\}\\ \;\;\;\;\;\;\;\;\;\;\;\;\;\;\;\;\;\;\;\;\;\;\;\;\;\;\;\;\;\;\;\;\;\;\;\;\;\;\;\;\;\;\;\;\;\;\;s\left(\tau -\frac{2R\left(p,q,t\right)}{c}\right)dpdq \end{array},$$

where t and τ are the time in azimuth and range, respectively, p and q are the azimuth and range position of the target, respectively, C is the observed area, x(·) is the backscattered coefficient of target, $\omega_{a}\left(\cdot \right)$ is the azimuth antenna pattern weighting, v is the platform speed, c is the speed of light, s(·) is the transmitted signal with carrier frequency $f_{c}$, and R(p,q,t) is the slant range.

Let $X\in {\mathbb{C}}^{N_{P}\times N_{Q}}$ denote the two-dimensional (2D) backscattering coefficients matrix, $x=\mathrm{vec}(X)\in {\mathbb{C}}^{N\times 1}(N=N_{P}N_{Q})$ is the vector of X reshaped sequentially column by column. The *n*-th element of x is $x(p_{n},q_{n})$. The time series is discretized into $T_{m}(m=1,2,\ldots ,M)$. Let $Y\in {\mathbb{C}}^{N_{t}\times N_{\tau }}$ denote the 2D echo data, and $y=\mathrm{vec}(Y)\in {\mathbb{C}}^{M\times 1}(M=N_{t}N_{\tau })$. Considering thermal noise $n\in {\mathbb{C}}^{M\times 1}$, then the sparse SAR imaging model can be assumed as [4]:

(2) y=Φx+n,

where $\Phi ≜\{\phi (m,n)\}\in {\mathbb{C}}^{M\times N}$ is the observation matrix, which represents the imaging geometry relationship between radar and surveillance regions, and can be written as

(3) $$\begin{array}{ll} \phi \left(m,n\right)≅ & \iint_{{}_{\left(t,\tau \right)\in T_{m}}}\omega_{a}\left(t-\frac{p_{n}}{v}\right)\exp\left\{-j4\pi f_{c}\frac{R\left(p_{n},q_{n},t\right)}{c}\right\}\\ & s\left(\tau -\frac{2R\left(p_{n},q_{n},t\right)}{c}\right)d\tau dt \end{array}$$

Considering data down-sampling, Equation (2) can be rewritten as:

(4) y=HΦx+n,

where H is the down-sampling matrix. Specifically, in a sparse SAR azimuth ambiguity suppression model, the echo data can be regarded as [24]:

(5) $$y=\left(\Phi_{-1},\Phi_{0},\Phi_{+1}\right)\left(\begin{array}{c} x_{-1}\\ x_{0}\\ x_{+1} \end{array}\right)+n=\Phi x+n,$$

where $x_{0}$, $x_{-1}$ and $x_{1}$ are the complex images measured by the radar mainlobe and sidelobe beams with different squint angles, and $\Phi_{i}(i=-1,0,1)$ are their observation matrices. When the observed scene is sufficiently sparse and the observation matrix satisfies the restricted isometry property (RIP) [25], Equation (4) can be solved by the $l_{q}(0<q\leq 1)$ regularization [26]:

(6) $$\hat{x}=\underset{x}{\arg\min}\{{\left\|y-H\Phi x\right\|}_{2}^{2}+\lambda {\left\|x\right\|}_{q}^{q}\},$$

where λ is the regularization parameter, which is determined by the scene sparsity. Equation (6) can be solved by convex optimization algorithms [27], nonconvex optimization algorithms [28], greedy tracking algorithms [29], Bayesian reconstruction algorithms [30] and so on. In our paper, IST is selected as an example to solve the sparse imaging model. After resolving $\hat{x}$, we need rearrange it into a matrix. 

### 2.1. Raw Data-based Sparse SAR Imaging

The azimuth-range decouple imaging method is one of RD-SIM, which introduces the echo simulation operator G(·) to replace the observation matrix Φ, which is the inverse operation of the MF imaging operator I(·), i.e., $G(\cdot )=I^{-1}(\cdot )\approx \Phi$. Then, the 2D sparse SAR imaging model can be written as [4,14]:

(7) $$Y=H_{a}G(X)H_{r}+N,$$

where Y is raw data, $H_{a}$ and $H_{r}$ are the azimuth and range down-sampling matrix, respectively, and N is the noise matrix. The optimization problem (6) can be rewritten as

(8) $$\hat{X}=\underset{X}{\arg\min}\{{\left\|Y-H_{a}G(X)H_{r}\right\|}_{F}^{2}+\lambda {\left\|X\right\|}_{q}^{q}\},$$

where ${\left\|\cdot \right\|}_{F}$ is the Fibonacci norm of a matrix. By using the iterative soft thresholding (IST) algorithm [31], Equation (8) can be solved iteratively,

(9) $$X^{\left(k+1\right)}=\eta_{\lambda ,\mu ,q}\left(X^{\left(k\right)}+\mu I\left(Y-H_{a}G\left(X^{\left(k\right)}\right)H_{r}\right)\right),$$

where μ is the iterative parameter, and $\eta_{\lambda ,\mu ,q}(\cdot )$ is the threshold function. When q=1, it can be defined as

(10) $$\eta_{\lambda ,\mu ,1}(x)=\frac{x}{\left|x\right|}\max(\left|x\right|-\frac{\mu \lambda }{2},0).$$

#### 2.2. Complex Image-based Sparse SAR Imaging

Different from RD-SIM, the input of this method is the complex image constructed by MF from raw data. Then, the feature-enhanced SAR images can be obtained by solving an $l_{q}$ regularization problem. In the following, CI-SIM is introduced in full- and under-sampling cases, respectively [23].

#### 2.2.1. Fully Sampled Data

For the fully sampled data, $H_{a}$ and $H_{r}$ are both identical matrices. After performing the imaging operator on I(·) for Equation (7), we have [32]

(11) $$\begin{array}{l} I(Y)=I(H_{a}G(X)H_{r})+I(N)\\ \Leftrightarrow X_{MF}=X+N\prime \end{array},$$

where $X_{MF}$ is the complex image obtained by MF from fully sampled raw data, that is, the algorithm input, and N′ represents the difference between the real scene X and the complex image, including noise, clutter and sidelobes. Then, according to the imaging model shown in Equation (11), we can reconstruct the observed scene by solving the $l_{q}(0<q\leq 1)$ optimization problem.

(12) $$\hat{X}=\underset{X}{\arg\min}\{{\left\|X_{MF}-X\right\|}_{F}^{2}+\lambda {\left\|X\right\|}_{q}^{q}\}$$

Similarly, using the IST algorithm, the observed scene can be recovered iteratively as [32]:

(13) $$X^{\left(k+1\right)}=\eta_{\lambda ,\mu ,q}\left(X^{\left(k\right)}+\mu (X_{MF}-X^{\left(k\right)})\right).$$

Compared with Equation (9), it is found that CI-SIM and RD-SIM are equivalent when the full-sampled data are available. Because each step of the iteration is based on the image, the imaging and inverse imaging operations in the complex image-based method can be omitted. Furthermore, the computational complexity can be reduced from O(2KMlogM+KN) to O(MlogM+KN), where *K* represents the number of iteration steps, and *M* and *N* denote the number of points in the raw data and discretized scene of interest, respectively.

#### 2.2.2. Under-Sampled Data

Under-sampling can be defined as the condition that the sampling rate is lower than the Nyquist sampling rate. There are several cases of under-sampled data in sparse SAR imaging: (1) down-sampling, making the average sampling rate lower than the Nyquist sampling rate; (2) nonuniform displace phase center sampling; and (3) sparse SAR imaging model-based azimuth ambiguity suppression. Similarly, taking down-sampled data as an example, the imaging model can be written as:

(14) $$\begin{array}{l} I(Y)=I(H_{a}G(X)H_{r})+I(N)\\ \Rightarrow X_{MF-U}=I(H_{a}G(X)H_{r})+N\prime \end{array},$$

where $X_{MF-U}$ is the complex image reconstructed by MF from under-sampled data. In this case,

(15) $$\begin{array}{c} X_{MF-U}\neq X_{MF}\\ I\left(H_{a}G\left(X\right)H_{r}\right)\neq X \end{array}.$$

Due to the under-sampling of raw data, a well-performing image cannot be obtained by the conventional MF algorithm. Besides, after performing down-sampling for the echo simulation operator, the observed scene cannot be recovered using the imaging operator I(·). Furthermore, the CI-SIM does not equal the RD-SIM.

## 3. Sparse SAR Imaging with Full- and Under-Sampling 

### 3.1. Full-Sampling

The sampling rate of fully sampled data is greater than or equal to the Nyquist sampling rate. The observed scene can be completely reconstructed by the MF method based on fully sampled data. For the fully sampled data, H is the identity matrix and can be ignored. The sparse SAR imaging model is shown in Equation (2). We have

(16) $$\Phi \in {\mathbb{C}}^{M\times N},M>N.$$

If we select the chirp scaling algorithm [33] as the imaging method, the MF operator I(·) and inverse operator of MF G(·) can be respectively expressed as:

(17) $$I\left(Y\right)=F_{a}^{-1}\left(F_{a}Y⊙\Theta_{sc}F_{r}⊙\Theta_{rc}F_{r}^{-1}⊙\Theta_{ac}\right),$$

(18) $$G\left(X\right)=F_{a}^{-1}\left(F_{a}X⊙\Theta_{ac}^{*}F_{r}⊙\Theta_{rc}^{*}F_{r}^{-1}⊙\Theta_{sc}^{*}\right),$$

where $\Theta_{sc}$ is the differential range cell migration correction (RCMC) matrix, $\Theta_{rc}$ is the range compression and bulk RCMC matrix, $\Theta_{ac}$ is the azimuth compression and phase correction matrix, $F_{a}$ and $F_{r}$ are the azimuth and range Fourier-transform operators, respectively, and ${\left(\cdot \right)}^{*}$ is the conjugate operator. In this case, the sparse imaging methods based on raw data and complex images have similar performance, because $G=I^{-1}$. They both can effectively suppress the noise and sidelobes, hence improving the image quality compared with the MF method.

### 3.2. Down-Sampling

Down-sampling includes uniform down-sampling and random down-sampling in the range or/and azimuth directions. For different down-sampling modes, the form of matrix H is different, and no longer the identity matrix.

Considering down-sampling, the sparse SAR imaging model is shown in Equation (4), and we have

(19) $$H\Phi \in {\mathbb{C}}^{L\times N},L<<N.$$

The MF-based method directly based on down-sampling data could result in degraded imaging performance, such as sidelobe rising and ambiguous targets. As shown in Equation (15), $X_{MF-D}$ is not equal to $X_{MF}$, and its performance is worse than $X_{MF}$. If the poor-performance complex image $X_{MF-D}$ is taken as the input, CI-SIM obviously cannot obtain a well-performing image [23]. The RD-SIM has the ability to image with less data when the sparsity of the observed scene and signal-to-noise ratio (SNR) satisfy certain conditions. Its performance is better than the CI-SIM in this case. 

### 3.3. Nonuniform Displace Phase Center Sampling

**Figure 1:**
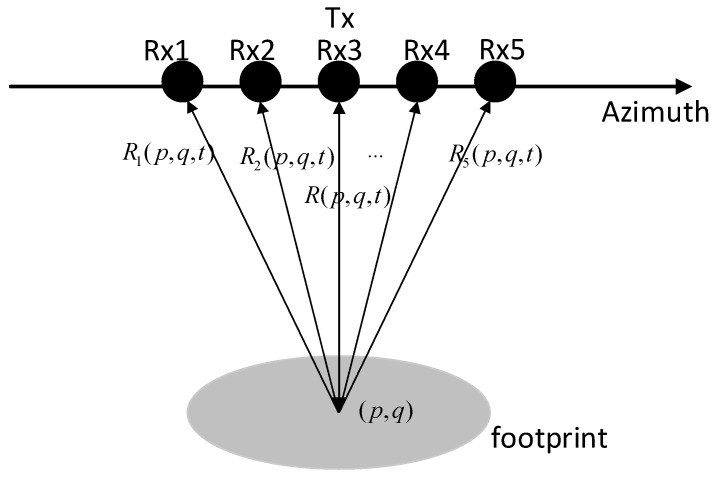
Single-transmit–multiple-receive multiple-channel synthetic aperture radar mode. Black circles correspond to transmitter (Tx) and receiver (Rx) positions.

The displaced phase center antenna (DPCA) technology was proposed to achieve high-resolution and wide-swath imaging in single-transmit–multiple-receive multiple-channel SAR mode [34], which is shown in Figure 1. The sparse imaging model at this point can still be represented by Equation (2), in which $y=[y_{1},y_{2},\ldots ,y_{I}]$, $y_{i}$ is the *i-*th channel data of multichannel SAR, I is the number of channels, $\Phi =[\Phi_{1},\ldots ,\Phi_{I}]$, and $\Phi_{i}$ is the *i-*th channel observation matrix whose elements are

(20) $$\begin{array}{ll} \Phi_{i}\left(m,n\right)≅ & \iint_{{}_{\left(t,\tau \right)\in T_{m}}}\omega_{a}\left(t-\frac{p_{n}}{v}\right)\exp\left\{-j2\pi f_{c}\frac{R\left(p_{n},q_{n},t\right)+R_{i}\left(p_{n},q_{n},t\right)}{c}\right\}\\ & s\left(\tau -\frac{R\left(p_{n},q_{n},t\right)+R_{i}\left(p_{n},q_{n},t\right)}{c}\right)d\tau dt \end{array},$$

where $R_{i}(p,q,t)=R(p,q,t-\frac{\Delta x_{i}}{v})$ represents the distance from the *i*-th receiver to the target.

In order to ensure uniform azimuth sampling, pulse repetition frequency (PRF) must satisfy

(21) $$\mathrm{PRF}=\frac{2v}{I\Delta x},$$

where Δx is the phase offset relative to the transmit aperture. The DPCA radar imaging system allows for an unambiguous recovery of the Doppler spectrum even for a nonuniform sampling of the SAR signal [35]. The DPCA technology-based single-transmit–multiple-receive multiple-channel SAR imaging operator I(·) and echo simulation operator G(·) can be respectively expressed as

(22) $$I\left(Y\right)=F_{a}^{-1}\left(\sum_{i}\left(F_{a}Y_{i}⊙P_{i}\right)⊙\Theta_{sc}F_{r}⊙\Theta_{rc}F_{r}^{-1}⊙\Theta_{ac}\right),$$

(23) $$G_{i}(X)=F_{a}^{-1}\left(F_{a}X⊙\Theta_{ac}^{*}F_{r}⊙\Theta_{rc}^{*}F_{r}^{-1}⊙\Theta_{sc}^{*}⊙P_{i}^{*}\right),$$

where $P_{i}$ is the reconstruction filter matrix. When the degree of nonuniform sampling is serious, there will still be tiny amounts of azimuth ambiguities in the image after using the matched filter bank to reconstruct the spectrum. If this complex image is used as input, the imaging performance will be decreased. A sparse signal processing technique has been applied to nonuniform displace phase center sampling SAR imaging, which is capable of suppressing ambiguity and is meanwhile insensitive to additive noise [19].

### 3.4. Sparse SAR Imaging Model-based Azimuth Ambiguity Suppression

The spectrum of the SAR antenna beam is not band-limited. Since the spectrum repeats at PRF intervals, the signal components outside this frequency interval fold back into the main part of the spectrum, which will lead to azimuth ambiguities. Azimuth ambiguities are a critical issue in an SAR system, especially in spaceborne SAR. Strong ambiguous signals can cause false alarms in the radar image, which affect SAR image interpretation. To suppress azimuth ambiguities, the echo data can be regarded as Equation (5). It is worth noting that the PRF at this time is less than the azimuth bandwidth. Considering the azimuth direction only, the elements of the *k*-th ambiguity area observation matrix $\Phi_{k}$ are as follows:

(24) $$\phi_{k}(m,n)=\int_{t\in T_{m}}\omega_{a}\left(t-\frac{p_{n}}{v}\right)\exp\left\{-j2\pi \frac{{\left(vt-p_{n}\right)}^{2}}{\lambda R}\right\}\exp(j\cdot k\mathrm{PRF}t)dt.$$

In the above azimuth ambiguity suppression model, azimuth ambiguities can be suppressed effectively because the reflectivity of the target is extended as the group sparse signal, and its components are jointly recovered by an $l_{q}$ regularization method based on raw data [4,24]. However, the ability of azimuth ambiguity suppression is limited by the CI-SIM.

## 4. Experiments

To compare imaging performance of the RD-SIM and CI-SIM under full-sampling and three under-sampling cases, the experiments are carried out with Radarsat-1 data, Gotcha Volumetric SAR data and C-band airborne data. The RadarSat-1 data was acquired on 16 June 2002. The acquisition mode is Fine, which covers an area of 50 km × 50 km with a resolution of 10 m. PRF is 1257 Hz**.** Radar frequency is 5.3 GHz. The Gotcha Volumetric SAR dataset consists of SAR phase history data collected at X-band with a 640 MHz bandwidth with full azimuth coverage at eight different elevation angles and full polarization. The C-band airborne data was acquired by the Institute of Electronics, Chinese Academy of Sciences, with standard stripmap mode. There are many algorithms for sparse signal processing. In our experiments, IST is selected as the recovery algorithm for both methods.

### 4.1. Full-Sampling

**Figure 2:**
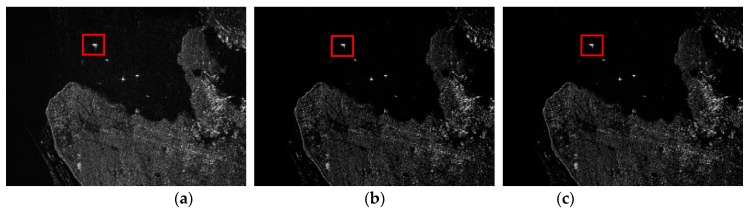
Images recovered from fully sampled Radarsat-1 data via different methods. (Red square indicates one ship. (**a**) Matched filtering. (**b**) Raw data-based sparse imaging method. (**c**) Complex image-based sparse imaging method.

The images recovered from the fully sampled Radarsat-1 raw data via MF, the RD-SIM and the CI-SIM are shown in Figure 2. The observed region is the University of British Columbia. Figure 2 shows that all three imaging methods can construct the region well with fully sampled echo data. The reconstructed results of the RD-SIM and CI-SIM are similar and have a better performance compared with the MF-constructed image because of lower sidelobes and less noise in the observed area.

In order to quantitatively evaluate the effects of different algorithms in suppressing image background clutter and noise, target-to-background ratio (TBR) is used as an evaluation indicator whose definition is shown as [36]:

(25) $$\mathrm{TBR}(X{)=20\log}_{10}\left(\frac{\max_{(p,q)\in T}\left|X_{\left(p,q\right)}\right|}{1/N_{B}\sum_{(p,q)\in B}\left|X_{\left(p,q\right)}\right|}\right),$$

where T is the target region, B is the background region, and $N_{B}$ is the number of pixels in B. One ship shown in the red frame is selected as a performance test area. The TBRs of the selected areas shown in Figure 2a–c is 45.13 dB, 52.39 dB and 52.39 dB, respectively. Obviously, the performance of sparse imaging methods based on raw data and complex images is superior to MF.

**Figure 3:**
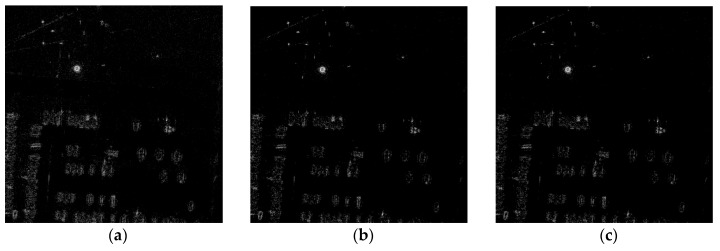
Images recovered from fully sampled X-band Gotcha Volumetric SAR data via different methods. (**a**) Matched filtering. (**b**) Raw data-based sparse imaging method. (**c**) Complex image-based sparse imaging method.

**Figure 4:**
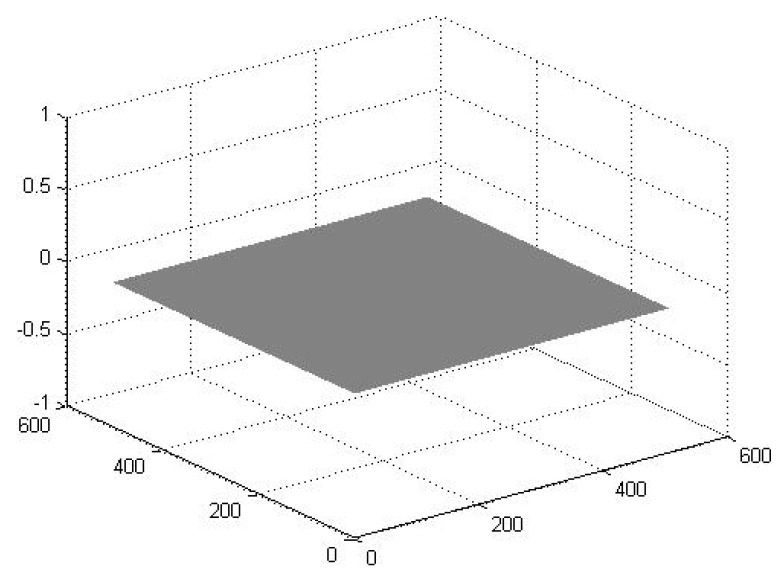
The difference between the recovered complex images of RD-SIM and CI-SIM.

As for the X-band Gotcha Volumetric SAR data, the recovered images via MF, the RD-SIM and the CI-SIM are shown in Figure 3. The imaging scene consists of numerous civilian vehicles and calibration targets. Figure 4 is the difference between the recovered complex images of RD-SIM and CI-SIM. We can see that the value of each point in Figure 4 is almost equal to zero, which means that the RD-SIM and the CI-SIM are equivalent. 

### 4.2. Down-Sampling

**Figure 5:**
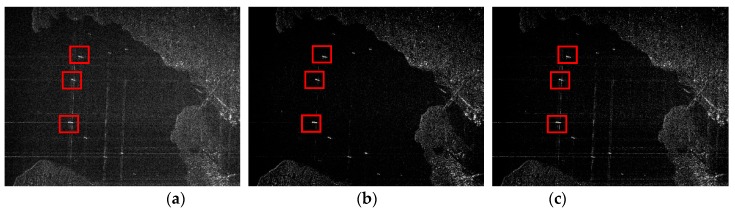
Images reconstructed from 80% down-sampled echo data by different methods. (Red squares indicate three ships.) (**a**) Matched filtering. (**b**) Raw data-based sparse imaging method. (**c**) Complex image-based sparse imaging method.

In this part, the English Bay ships’ region is selected as the region of interest (ROI). This region is a typical sparse scene that is convenient for comparing the performance of the two sparse imaging methods. The data come from Radarsat-1, and we perform 80% random down-sampling for the fully sampled Radarsat-1 raw data, which means that only 80% of the data is available. Figure 5 shows the recovered observed region from down-sampled echo data via MF, the RD-SIM and the CI-SIM. Due to the data down-sampling, it is obvious that MF could not recover the target successfully because of apparent energy dispersion in the azimuth and range directions. Similarly, the CI-SIM firstly needed to use MF to reconstruct a complex image, and then it was hardly able to achieve good performance based on the poor-performing complex image. However, the RD-SIM recovered the observed region successfully, and acquired an image with lower sidelobes and less noise. This experimental result shows that the complex image-based imaging method cannot process sparse imaging based on down-sampled echo data very well, compared to the raw data-based imaging method.

Three ships in the red frame are selected as the observed targets, and their TBRs reconstructed by three algorithms are shown in Table 1. It can be seen from Table 1 that the raw data-based sparse SAR imaging method performs better than the complex image-based sparse SAR imaging method.

### 4.3. Nonuniform Displace Phase Center Sampling

**Figure 6:**
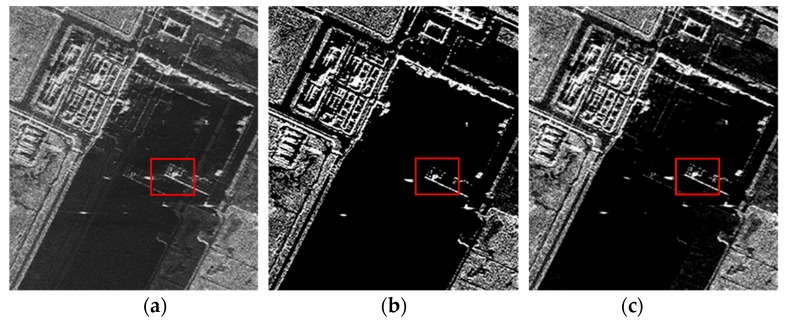
Image reconstructed via different algorithms with single-transmit three-receive SAR data. (**a**) Matched filtering. (**b**) Raw data-based sparse imaging method. (**c**) Complex image-based sparse imaging method.

The raw data used in the experiment are from the single-transmit three-receive SAR system simulated by the C-band airborne data of the Institute of Electronics of the Chinese Academy of Sciences through re-interpolation. The observed scene was a harbor in Tianjin, China. Experimental parameters are shown in Table 2. Multichannel data with different intersample offsets are resampled from the real data using the sinc interpolation method. Then, three methods are used to reconstruct the observation scene based on the multichannel data. The imaging results of each algorithm are shown in Figure 6. We can see the performance of different imaging algorithms on noise, sidelobes and azimuth ambiguity suppression. 

The ship in the red frame of the dock is selected as the observed area, and its TBRs are reconstructed by three algorithms, as shown in Table 3. It shows that the RD-SIM can reduce noise effectively, while the CI-SIM cannot achieve similar performance to the raw data-based sparse SAR imaging method.

### 4.4. Sparse SAR Imaging Model-based Azimuth Ambiguity Suppression

**Figure 7:**
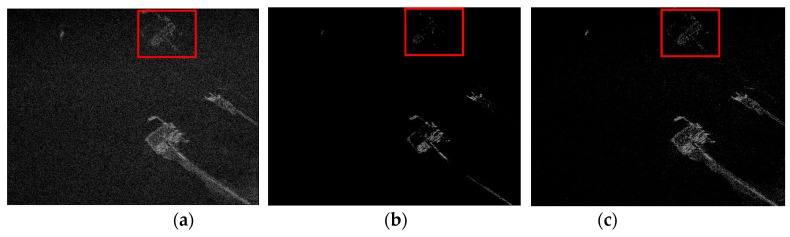
Azimuth ambiguity suppression via different algorithms. (**a**) Matched filtering. (**b**) Raw data-based sparse imaging method. (**c**) Complex image-based sparse imaging method.

To compare imaging performance of the RD-SIM and CI-SIM in azimuth ambiguity suppression, another coastal region is selected as the observed scene from the same RadarSat-1 dataset. According to Section 3, the azimuth spectrum will be aliased in limited PRF conditions due to the azimuth beam pattern. The recovered scenes are shown in Figure 7.

The azimuth ambiguity-to-signal ratio (AASR) is selected to measure the ability of different algorithms to suppress azimuth ambiguities, which is defined as:

(26) $$\mathrm{AASR}=10\log_{10}\left(\frac{\frac{1}{N_{m}}\sum_{(p,q)\in M_{a}}{\left|X_{\left(p,q\right)}\right|}^{2}}{\frac{1}{N_{a}}\sum_{(p,q)\in A}{\left|X_{\left(p,q\right)}\right|}^{2}}\right),$$

where $M_{a}$ is the ambiguity area, $N_{m}$ is the number of pixels in $M_{a}$, A is the target area and $N_{a}$ is the number of pixels in A. The AASR of the target via three algorithms is shown in Table 4. Obvious azimuth ambiguities in the imaging results reconstructed by MF are shown in the red frame of Figure 7a. Figure 7b and Table 4 show that azimuthal ambiguities are effectively suppressed by the RD-SIM. The performance of the CI-SIM is better than MF but worse than the RD-SIM.

## 5. Conclusions

In this paper, we compare the RD-SIM and the CI-SIM, and expound their relationship. It shows that the two methods are equivalent when the raw data is fully sampled. Meanwhile, the RD-SIM performs better than the CI-SIM when processing down-sampled data, and performing nonuniform displace phase center sampling, and sparse SAR imaging model-based azimuth ambiguity suppression. Obviously, when the data is fully sampled, the CI-SIM is the better choice because the computational complexity is reduced greatly. When the data is under-sampled, better imaging performance can be obtained based on the raw data. The conclusions are applicable to datasets with different polarization modes in different frequency bands, and are well proved via experiments in full- and under-sampling cases. 

## Figures and Tables

**Table 1:** Target-to-background ratio (TBR) of target area via different methods with down-sampled data.

Imaging Algorithm	Target-to-Background Ratio (TBR/dB)		
	Ship 1	Ship 2	Ship 3
**MF**	30.35	33.44	19.62
**RD-SIM**	49.14	50.59	43.26
**CI-SIM**	47.46	46.89	33.39

**Table 2:** Parameters.

Parameters	Value
**Carrier frequency**	5.4 GHz
**Velocity**	100 m/s
**Pulse duration**	38 µs
**Antenna length (Tx/Rx)**	0.9 m
**Sampling rate**	750 MHz
**PRF**	768 Hz
**Number of subapertures (Rx)**	3

**Table 3:** Target-to-background ratio (TBR) of target area via different algorithms with multichannel data.

Imaging Algorithm	Target-to-Background Ratio (TBR/dB)
**MF**	29.86
**RD-SIM**	55.37
**CI-SIM**	36.72

**Table 4:** The azimuth ambiguity-to-signal ratio (AASR) of the target via three algorithms.

Imaging Algorithm	Azimuth Ambiguity-to-Signal Ratio (AASR/dB)
**MF**	−22.86
**RD-SIM**	−34.77
**CI-SIM**	−28.72
